# Dual PI3K/AKT and CDK4/6 inhibition reveals selective sensitivity in an SHH medulloblastoma stem cell model

**DOI:** 10.1002/1878-0261.70230

**Published:** 2026-02-24

**Authors:** Monika Lukoseviciute, Madeleine Birgersson, Paolo Ceriani, Margareta Wilhelm, Ourania N Kostopoulou

**Affiliations:** ^1^ Department of Oncology‐Pathology Karolinska Institutet Stockholm Sweden; ^2^ Department of Microbiology, Tumor and Cell Biology Karolinska Institutet Stockholm Sweden

**Keywords:** AKT inhibitors, CDK4/6 inhibitors, Medulloblastoma, Neuroepithelial stem cells, PI3K inhibitors, targeted therapy

## Abstract

Medulloblastoma (MB) is a brain tumor for which current treatments cause serious side effects and are not curative for all patients, highlighting the need for more effective and brain‐protecting therapies. Recently, we combined phosphoinositide 3‐kinase (PI3K) inhibitor (BYL719), fibroblast growth factor receptor (FGFR) inhibitor (JNJ‐42756493) and cyclin‐dependent kinase (CDK)4/6 inhibitor (PD‐0332991) in MB cell lines, and discovered synergistic effects. In the current study, we investigate the most efficient therapies in a normal/tumorigenic neural stem cell model. A sonic hedgehog (SHH)‐MB model, including a Gorlin syndrome patient neuroepithelial stem cell line (NES) and its tumor derivative (tNES), was used to evaluate single and combined treatments of PI3K, AKT, FGFR, and CDK4/6 inhibitors (BYL719, AZD5363, JNJ‐42756493, and PD‐0332991, respectively). Effects on viability, cell confluence and apoptosis were tested on NES and tNES cells cultured as 2D monolayers and 3D spheroids. We found that 2D tNES cells were generally more sensitive to the inhibitory effects of both single and combination treatments compared to 2D NES cells. In the 3D setting, all single drugs were more effective against tNES than NES, except for JNJ‐42756493, which showed the opposite trend. Drug combinations in 3D cultures generally resulted in synergistic or additive effects on cell viability in NES and tNES. This study illustrates that single and combined administrations of PI3K, FGFR, CDK4/6, and AKT inhibitors in a NES/tNES model have dose‐dependent and additive/synergistic anti‐MB activity impacting tumor growth. Their effects on tNES cells were generally more pronounced than on NES; however, the difference in proliferative capacity between the cells should be considered.

## Graphical abstract

Targeted therapy was evaluated in SHH medulloblastoma using neuroepithelial stem cell (NES) and tumor‐derived NES‐like (tNES) models in 2D monolayers and 3D spheroids. PI3K, AKT, and CDK4/6 inhibitors had minimal effects in NES but markedly reduced viability and growth and induced apoptosis in tNES cells, revealing distinct therapeutic vulnerabilities.

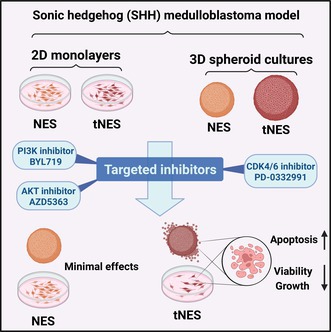

**Abbreviations**
2D: Two dimensional
3D: Three dimensional
AKT: Protein kinase B
ATP: Adenosine triphosphate
BSA: Bovine serum albumin
CC3: Cleaved caspase 3
CDK4/6: Cyclin‐dependent kinases 4 and 6
CT: Chemotherapy
DAB: 3,3′‐Diaminobenzidine
DMEM/F12: Dulbecco's modified Eagle medium/Nutrient Mixture F‐12
DMSO: Dimethyl sulfoxide
EGF: Epidermal growth factor
FDA: Food and Drug Administration
FGF2: Fibroblast growth factor 2
FGFR: Fibroblast growth factor receptor
GLI2: GLI family zinc finger 2
HAS: Highest single agent
HRP: Horse‐radish peroxidase
IHC: Immunohistochemistry
iPS: Induced pluripotent stem cells
MB: Medulloblastoma
NB: Neuroblastoma
NES: Neuroepithelial stem cells
o/n: Overnight
PBS: Phosphate‐buffered saline
PI3K: Phosphoinositide 3‐kinase
PTCH1: Patched 1
Rb: Retinoblastoma protein
RT: Room temperature
SD: Standard deviation
SHH: Sonic hedgehog
SMO: Smoothened
SUFU: Suppressor of fused
tNES: Tumor‐derived neuroepithelial stem cells
TP53: Tumor protein p53
WNT: Wingless‐related integration site
WST‐1: Water‐soluble tetrazolium salt‐1; cell viability assay

## 1 Introduction

Medulloblastoma (MB) is a highly aggressive childhood brain malignancy originating in the cerebellum, with significant molecular and clinical heterogeneity [1, 2]. Based on molecular profiles, MB is classified into four major subgroups: WNT‐activated, SHH‐activated (subdivided into TP53 wild‐type and TP53 mutant), and a non‐WNT/non‐SHH group, which includes Group 3 and Group 4 [2, 3]. The SHH‐driven subgroup accounts for approximately 30% of all MB cases and is marked by aberrant activation of the SHH signaling pathway, which is crucial for tumor cell proliferation and growth [2, 4, 5]. Dysregulated SHH signaling is often linked to mutations in key components such as PTCH1, SMO, SUFU, or GLI2 [6, 7, 8]. Notably, SHH‐MB is also frequently associated with cancer predisposition syndromes, particularly Gorlin syndrome, caused by germline mutations in PTCH1 or SUFU [9, 10].

Despite advances in treatment, including surgery, chemotherapy (CT), and radiotherapy (RT), current therapies cause long‐term side effects and often fail to specifically target the molecular mechanisms driving tumor growth, leading to treatment resistance [11, 12, 13, 14, 15, 16, 17, 18, 19]. In this context, applying various targeted therapies could offer a novel approach to improve treatment efficacy.

Multiple signaling pathways and tumor drivers are involved in MB tumor development and progression [20, 21]. The phosphoinositide 3‐kinase/protein kinase B (PI3K/AKT) pathway plays a critical role in MB cell survival, differentiation, proliferation, and metabolism, and mutations in the PI3K/AKT pathway are also found in MB [22, 23, 24, 25, 26]. Moreover, mutations in the catalytic subunit alpha of PI3K have been shown to enhance tumor growth and promote metastasis in SHH‐MB mouse models [27]. Alongside the PI3K/AKT pathway, cyclin‐dependent kinases (CDK) 4 and 6 are key regulators of the cell cycle and are often amplified in MB, leading to uncontrolled cell division [26, 28, 29]. Hence, the CDK4/6/cyclin D/Rb pathway has been proposed as a potential therapeutic target for all non‐WNT MB [30]. In addition, fibroblast growth factor receptor (FGFR) activation promotes invasiveness and cell growth in MB cells, making it an important therapeutic target [31, 32].

Previously, we targeted some of the above pathways and demonstrated that combinations of Food and Drug Administration (FDA) approved BYL719 (PI3K), with AZD5363 (AKT), PD‐0332991 (CDK4/6) or JNJ‐42756493 (FGFR) produced synergistic effects in MB cell lines [33, 34, 35, 36, 37].

Here, to compare the effects of targeted therapies under normalized vs. tumor‐like conditions, a pilot study was performed. More specifically, neuroepithelial stem (NES) cells derived from induced pluripotent stem (iPS) cells of a Gorlin syndrome patient with a germline *PTCH1* mutation and its tumor‐derived counterpart tNES from the same patient were used [38]. The tNES cells, which resemble SHH‐driven MB, were originally generated from tumors derived from NES cells transplanted into mouse cerebellum [38].

More specifically, the therapeutic effects of the PI3K, CDK4/6, and FGFR inhibitors mentioned above, along with the newly FDA‐approved AKT inhibitor AZD5363 (33), were tested both alone and in combination, in parallel on NES and tNES cells grown in two‐dimensional (2D) monolayer cultures and three‐dimensional (3D) spheroid cultures.

## 2 Materials and methods

### 2.1 Cells and culture conditions

Generation of NES and tNES cells has been described previously [38]. Both NES and tNES have been provided from Associate Prof. Margareta Wilhelm, Department of Microbiology, Tumor and Cell biology, Karolinska Institutet. They were both cultured as monolayers in flasks coated with 20 μg·mL^−1^ Poly‐L‐ornithine (Sigma‐Aldrich, Merck, Darmstadt, Germany) and 1 μg·mL^−1^ laminin (L2020, Sigma‐Aldrich, Merck) in DMEM/F12 medium (Gibco; Thermo Fisher Scientific, Waltham, MA, USA) containing 1% Penicillin–Streptomycin (Pen/Strep) (Gibco; Thermo Fisher Scientific), 1% N2 (Gibco; Thermo Fisher Scientific), 0.1% B27 (Gibco; Thermo Fisher Scientific), 10 ng·mL^−1^ FGF2 (Gibco; Thermo Fisher Scientific) and 10 ng·mL^−1^ EGF (PeproTech, Thermo Fisher Scientific, Rocky Hill, NJ, USA). All cells used in this study were routinely tested and confirmed to be mycoplasma free.

For monolayer (2D) growth, NES and tNES cells were seeded in 96‐well tissue culture plates coated with 20 μg·mL^−1^ Poly‐L‐ornithine (Sigma‐Aldrich, Merck) and 1 μg·mL^−1^ laminin (Sigma‐Aldrich, Merck) and for the viability assay they were seeded at 10000 cells in 90 μL medium/well.

To establish 3D cultures (spheroids) of 300–400 μm in diameter, 5000 and 3000 single‐cell suspensions of NES and tNES, respectively, were seeded in 100 μL medium in 96‐well, Nunclon Sphera‐Treated, U‐Shaped‐Bottom Microplates ULA (Thermo Fisher Scientific) and cultured for 72 h before drug treatment. The cultures were then followed for viability assay, measurement of spheroid growth, and immunohistochemistry (IHC).

### 2.2 Inhibitors

The FDA‐approved PI3K inhibitor BYL719 (alpelisib), AKT inhibitor AZD5363 (Capivasertib), CDK4/6 inhibitor PD‐0332991 (palbociclib), and FGFR inhibitor JNJ‐42756493 (erdafitinib) were all purchased from TargetMol (San Diego, California, USA). All initial stocks were prepared in DMSO and later diluted in PBS to each drug's working stock concentration (0.05–1 μm) after proceeding with initial drug titrations.

### 2.3 WST‐1 cell viability assay for 2D studies

To assess cell viability in 2D cultures, the WST‐1 assay (Roche Diagnostics, Mannheim, Germany) was used. This colorimetric method indicates viable cells and was performed according to the previously described protocol [39].

### 2.4 CellTiterGlo 3D cell viability assay

For 3D cultures, viability was measured with the CellTiterGlo reagent (Promega, Madison, WI, USA), according to the manufacturer's instructions. This method utilizes ATP quantification to mark metabolically active cells. Viability was measured 24, 48, and 72 h after treatment, where 100 μL CellTiterGlo reagent was added to each well at room temperature (RT), followed by 5 min of shaking. After 25 min incubation at RT, luminescence was measured using the Varioskan™ LUX multimode microplate reader (Thermo Fisher Scientific). The viability was calculated by averaging the luminescence, subtracting the blank, and normalizing to the PBS‐treated control.

### 2.5 Spheroid growth assayed by the Incucyte® S3 live‐cell analysis instrument

After the spheroid formation, as described in the section ‘Cells and culture conditions’ the spheroids were treated with our tested drugs (mentioned in the Section ‘Inhibitors’), and the Incucyte® S3 Live‐Cell Analysis Instrument (Sartorius, Göttingen, Germany) was used for live imaging. Images of each spheroid were captured every 2 h, and their size was calculated as the largest object area in each image using the Incucyte software 2024A.

### 2.6 Immunohistochemistry

Spheroids treated for 48 h and corresponding controls were collected from a 96‐well spheroid plate and fixed in 4% paraformaldehyde (Santa Cruz Biotechnology, Dallas, TX, USA) overnight (o/n) at 4 °C. After changing to 70% ethanol, the samples were stored at 4 °C. The fixed spheroids were dehydrated in an automated tissue processing machine (LOGOS, Milestone Srl, Sorisole, Bergamo, Italy), embedded in paraffin, cut into 4 μm thick sections, and mounted on glass slides (Superfrost, Thermo Fisher Scientific, Waltham, MA, USA). All slides were heated at 56 °C for 3 h.

Before staining, the slides were deparaffinized in xylene and rehydrated in decreasing concentrations of ethanol. Heat‐induced epitope retrieval was performed in Citrate buffer pH 6 (Sigma‐Aldrich, Merck, C‐9999, Schnelldorf, Germany) using a Decloaking Chamber (Biocare Medical, Pacheco, California, USA) set for 5 min at 110 °C. Endogenous peroxidase activity was blocked by incubation with 0.15% hydrogen peroxide for 30 min at RT. Further blocking with Biocare Medical Background Sniper (Biocare Medical, Pacheco, California, USA) for 15 min was done to reduce non‐specific background staining. The tissue sections were incubated o/n at 4 °C in a humidity chamber with the primary antibodies, anti‐Ki67 (1 : 800, DAKO, #A0047, Lot nr, RRID, UK Ltd., Cambridgeshire, UK) to assess proliferation or anti‐cleaved caspase 3 (anti‐CC‐3) (1 : 2000, Cell Signaling, #9661, Lot nr, RRID: AB_2341188 Danvers, MA, USA) to assess apoptosis. Both antibodies were diluted in PBS with 1% bovine serum albumin (BSA). Detection was done using the Bio Biocare Medical Mach‐1 Universal Horse‐Radish‐Peroxidase (HRP)‐Polymer Kit (MIU539L10, Biocare Medical, Pacheco, California, USA) according to the manufacturer's protocol and developed for 5 min with 3,3′‐diaminobenzidine (DAB) (Sigma‐Aldrich, Merck, Schnelldorf, Germany). After counterstaining with Mayer's hematoxylin (Sigma‐Aldrich, Merck) for 1 min, the sections were dehydrated in an increasing gradient of ethanol and xylene and then mounted using Pertex (Histolab, Gothenburg, Sweden). The images were obtained using the Olympus BH‐2 microscope (Tokyo, Japan) and DMK 33UX264 series monochrome industrial camera (The Imaging Source, Bremen, Germany).

### 2.7 Synergy calculation and statistical analysis

The effectiveness of the drug combinations was assessed using the Synergy FinderPlus computational tool (https://synergyfinderplus.org/), with the highest single agent (HSA) approach. HSA values greater than 10 indicate synergistic effects of the drugs, values between −10 and 10 suggest additive effects, and values less than −10 indicate antagonism [40, 41]. All experiments were repeated at least three times and are depicted as mean values with standard deviations (SD). The difference between the combinational treatments and the corresponding single treatments was estimated using two‐way ANOVA with Dunnett's multiple comparisons test, *P* < 0.05 was considered significant. All reported *P* values are referred to 72 h unless they are specified.

## 3 Results

### 3.1 Impact of single and combined FGFR, PI3K, CDK4/6, and AKT inhibitors on cell viability in 2D cultures of NES and tNES cells (i.e., 2D NES and 2D tNES)

#### 3.1.1 Single treatments on 2D NES cells

**Fig. 1:**
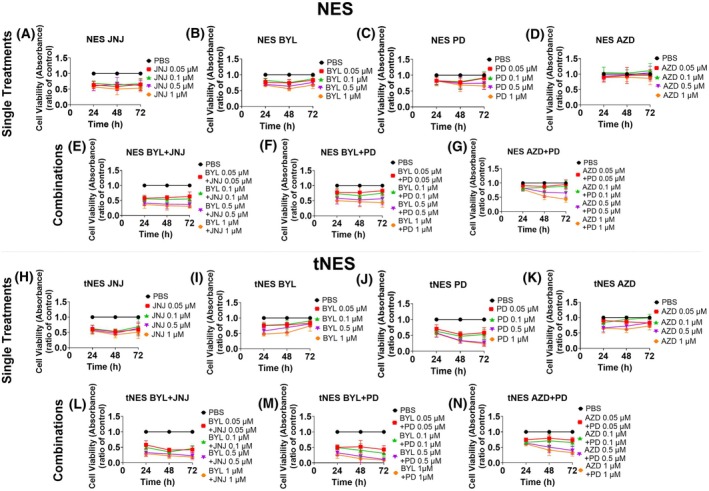
Impact of single and combined treatments with FGFR, PI3K, CDK4/6, and AKT inhibitors JNJ‐42756493, BYL719, PD‐0332991, and AZD5363, respectively, on the viability of NES and tNES monolayers. Cell viability of 2D NES (A–G) and tNES (H–N) cultures was assessed by absorbance measurements at 24, 48, and 72 h following treatment. Data represent independent biological replicates as follows: panel A, E, N, *n* = 3; panel B, D, F, (J), K, L, *n* = 5; panels C, *n* = 7; panels G, (I), *n* = 4; panels H, (M), *n* = 6. Error bars indicate standard deviation (SD). NES, neuroepithelial stem cells; tNES, tumor‐derived neuroepithelial stem cells; FGFR, fibroblast growth factor receptor; PI3K, phosphoinositide 3‐kinase; CDK4/6, cyclin‐dependent kinases 4 and 6; AKT, protein kinase B; JNJ, JNJ‐42756493; BYL, BYL719; PD, PD‐0332991; AZD, AZD5363.

Although all 0.05–1 μm doses of the FGFR inhibitor JNJ‐42756493 inhibited NES cell viability up to 50% only the highest dose showed significant effect (*P* < 0.05) (Fig. 1A). PI3K and CDK4/6 inhibitors, BYL719 and PD‐0332991 respectively, significantly decreased cell viability in the highest doses (*P* < 0.05). (Fig. 1B,C). In contrast, similar doses of the AKT inhibitor (AZD5363) had almost no effect on cell viability (Fig. 1D).

#### 3.1.2 Single treatments on 2D tNES

Notably, almost all tested doses (0.05–1 μm) of the FGFR and CDK4/6 inhibitors, JNJ‐42756493 and PD‐0332991 respectively, significantly decreased tNES viability (*P* < 0.05) (Fig. 1H,J) while the PI3K and AKT inhibitors BYL719 and AZD5363, respectively, had more limited effects (Fig. 1I,K).

#### 3.1.3 Combined treatments on 2D NES cells

Combining the various inhibitors had moderate effects on NES cell viability. Most of the combinations of BYL719 and JNJ‐42756493 significantly decreased NES cell viability (*P* < 0.05) (Fig. 1E). A 50% decrease in NES viability was only obtained with the two highest drug doses, that is, 0.5 μm and 1 μm of the BYL719 and PD‐0332991 combination (*P* < 0.05) (Fig. 1F), while for the AZD5363 and PD‐0332991 combination this was only obtained with the highest drug dose 1 μm (*P* < 0.05) (Fig. 1G).

#### 3.1.4 Combined treatments on 2D tNES cells

All 0.05–1 μm BYL719 and JNJ‐42756493, BYL719 and PD‐0332991, as well as AZD5363 and PD‐0332991 combinations significantly decreased the tNES viability by at least 50% compared to the PBS control, except for the two lower concentrations (0.05 μm and 0.1 μm) of the AZD5363 and PD‐0332991 combination (*P* < 0.05) (Fig. 1L–N).

To summarize, tNES cells were generally more sensitive than NES cells to both single and combination treatments in 2D cultures.

### 3.2 Impact of single and combined FGFR, PI3K, CDK4/6, and AKT inhibitors on cell viability in 3D cultures of NES and tNES cells (i.e., 3D NES and 3D tNES)

#### 3.2.1 Single treatments on 3D NES

**Fig. 2:**
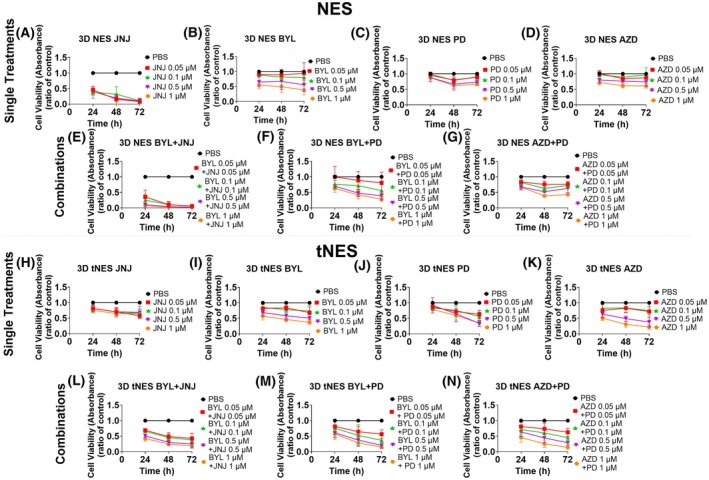
Impact of single and combined treatments with FGFR, PI3K, CDK4/6, and AKT inhibitors JNJ‐42756493, BYL719, PD‐0332991, and AZD5363, respectively, on the viability of NES and tNES spheroids. Cell viability of NES (A–G) and tNES (H–N) spheroids was assessed by luminescence measurements at 24, 48, and 72 h following treatment. Data represent independent biological replicates as follows: panels A, B, C, E, L, N, *n* = 4; panels D, H, J, M, *n* = 5; panels F, G, *n* = 3; panels I, K, *n* = 6. Error bars indicate standard deviation (SD). NES, neuroepithelial stem cells; tNES, tumor‐derived neuroepithelial stem cells; FGFR, fibroblast growth factor receptor; PI3K, phosphoinositide 3‐kinase; CDK4/6, cyclin‐dependent kinases 4 and 6; AKT, protein kinase B; JNJ, JNJ‐42756493; BYL, BYL719; PD, PD‐0332991; AZD, AZD5363.

In 3D NES, JNJ‐42756493 had the most pronounced impact among the single treatments, like 2D NES, with all tested doses 0.05–1 μm significantly reducing cell viability by more than 50% at all time points (*P* < 0.005) (Fig. 2A). BYL719 also inhibited cell viability by more than 50% at the highest concentration 1 μm compared to the PBS control (*P* < 0.05) (Fig. 2B). 3D NES, showed however similar to 2D only moderate to mild reductions in cell viability upon AZD5363 and PD‐0332991 single treatments (Fig. 2C,D).

#### 3.2.2 Single treatments on 3D tNES

All drugs showed a measurable impact on the cell viability of 3D tNES spheroid after 72 h of treatment, with more pronounced effects observed at higher concentrations (*P* < 0.05) (Fig. 2H–K). Moreover, while the treatments generally had stronger effects on 3D tNES than on 3D NES, JNJ‐42756493 showed weaker cell viability reduction in 3D tNES, whereas BYL719 exhibited comparable efficacy across both models (Fig. 2A–D,H–K).

#### 3.2.3 Combined treatments on 3D NES and tNES

The combinations of 0.05–1 μm BYL719 with JNJ‐42756493 considerably decreased cell viability in 3D NES and 3D tNES, with 3D NES showing greater sensitivity (Fig. 2E,L).

Most of the tested combination doses of BYL719 with PD‐0332991 produced a similar trend in both 3D NES and 3D tNES, considerably reducing cell viability compared to the PBS control at both 48 and 72 h, with a more pronounced effect observed in 3D tNES (*P* < 0.005) (Fig. 2F,M).

Likewise, 0.05–1 μm combination doses of AZD5363 and PD‐0332991 were generally more effective in 3D tNES, with a marked reduction in viability in the latter observed at 48 and 72 h (*P* < 0.05) (Fig. 2G,N).

To summarize, all single‐drug treatments, except for JNJ‐42756493, tended to be more effective on 3D tNES compared to 3D NES. Moreover, combination treatments generally enhanced the effects on viability in both 3D NES and 3D tNES; however, also here JNJ‐42756493 exhibited stronger viability reduction in 3D NES than in 3D tNES.

### 3.3 Synergistic effects of inhibitor combinations in 3D NES and 3D tNES

**Fig. 3:**
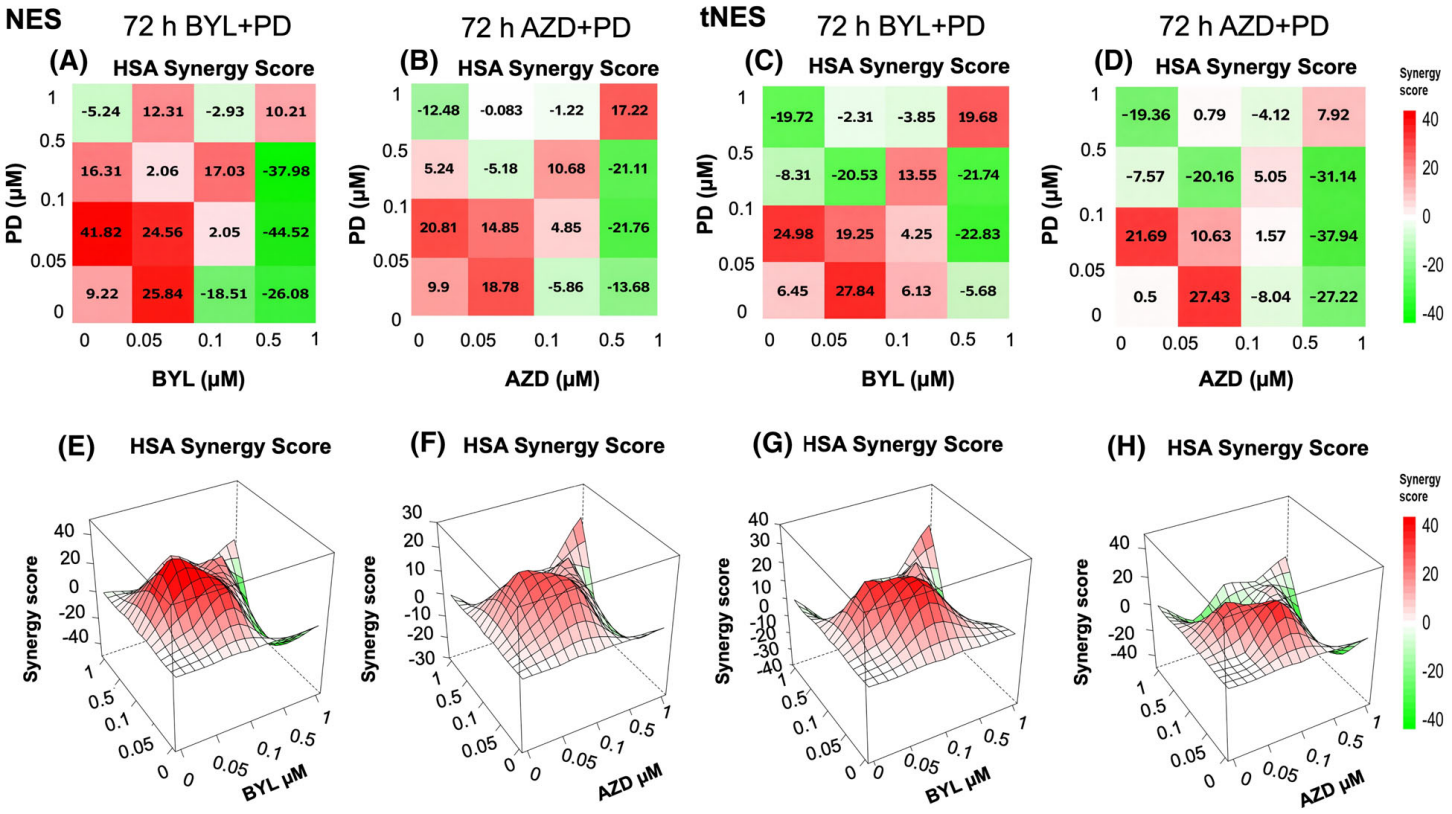
Evaluation of drug interactions between BYL719, PD‐0332991, and AZD5363 in NES and tNES spheroids 72 h after treatment. Drug interaction analyses were performed in NES and tNES spheroids using the SynergyFinder Plus platform. Heatmap plots (A–D) and 3D surface maps (E–H) display the interaction profiles and optimal combination doses of BYL719, PD‐0332991, and AZD5363 based on the Highest Single Agent (HSA) model. The synergy score reflects the interaction: values greater than 10 indicate a synergistic effect, values between −10 and 10 suggest an additive effect, and values below −10 indicate an antagonistic effect. NES, neuroepithelial stem cells; tNES, tumor‐derived neuroepithelial stem cells; BYL, BYL719; PD, PD‐0332991; AZD, AZD5363.

The synergistic potential of BYL719, AZD5363, and PD‐0332991 combinations was further evaluated in 3D NES and 3D tNES using 3D cell viability data (Fig. 3). Here, we aimed to determine whether significant differences existed between 3D NES and 3D tNES regarding the synergistic effects of the treatments. However, since JNJ‐42756493 alone and combined with BYL719 were highly effective, causing rapid cell death in 3D NES, the latter combination was not further investigated in detail, or compared with regard to its effects on 3D tNES.

Heatmaps and 3D surface plots, generated with the SynergyFinder Plus tool, were used to visualize drug interactions, with synergy quantified according to the Highest Single Agent (HSA) model (Fig. 3). In this model, HSA values greater than 10 indicate synergy, values between −10 and 10 indicate additive effects, and values below −10 represent antagonism.

In 3D NES, predominantly synergistic interactions were observed between BYL719 and PD‐0332991 as well as between AZD5363 and PD‐0332991, except for the lowest dose combinations (0.05 μm) which exhibited additive effects (Fig. 3A,B,E,F).

The same trend was observed in 3D tNES, with mostly synergistic effects between BYL719 and PD‐0332991, except at the lowest doses, which were additive (Fig. 3C,G). In comparison, the AZD5363 and PD‐0332991 combination mainly induced additive effects in tNES spheroids, with only one instance of synergy (Fig. 3D,H).

To summarize, BYL719 and PD‐0332991 combinations exhibited primarily synergistic effects in both 3D NES and 3D tNES, while AZD5363 and PD‐0332991 combinations had mainly synergistic effects in 3D NES and predominantly additive effects in 3D tNES.

### 3.4 Growth response of NES and tNES spheroids to PI3K, CDK4/6, and AKT inhibition

**Fig. 4:**
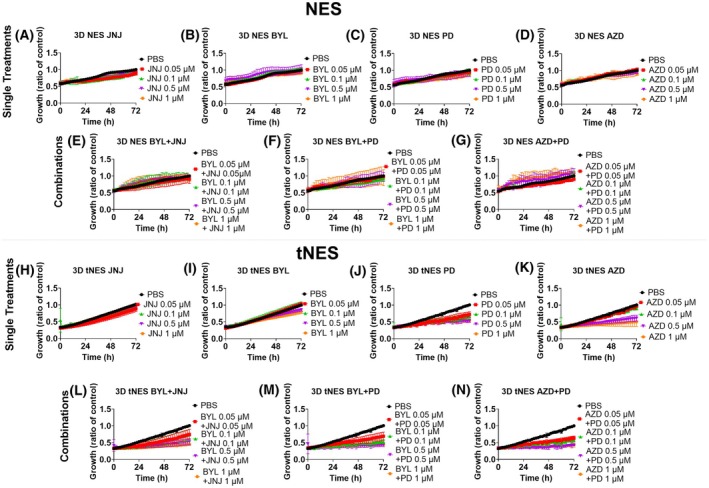
The efficacy of the FGFR, PI3K, CDK4/6, and AKT inhibitors JNJ‐42756493, BYL719, PD‐0332991, and AZD5363, respectively, alone and in combination, on the growth of NES and tNES spheroids. Growth of 3D NES (A–G) and 3D tNES (H–N) was monitored every 2 h for 72 h following treatment. Values were normalized to the PBS control within each experiment and treatment condition. Data represent independent biological replicates as follows: panels A, B, G, H, and I, *n* = 3; panels C, D, E, and F, *n* = 5; panels J, K, L, and M, *n* = 6; and panel N, *n* = 4. Error bars indicate the standard deviation of independent biological replicates. NES, neuroepithelial stem cells; tNES, tumor‐derived neuroepithelial stem cells; FGFR, fibroblast growth factor receptor; PI3K, phosphoinositide 3‐kinase; CDK4/6, cyclin‐dependent kinases 4 and 6; AKT, protein kinase B; JNJ, JNJ‐42756493; BYL, BYL719; PD, PD‐0332991; AZD, AZD5363.

**Fig. 5:**
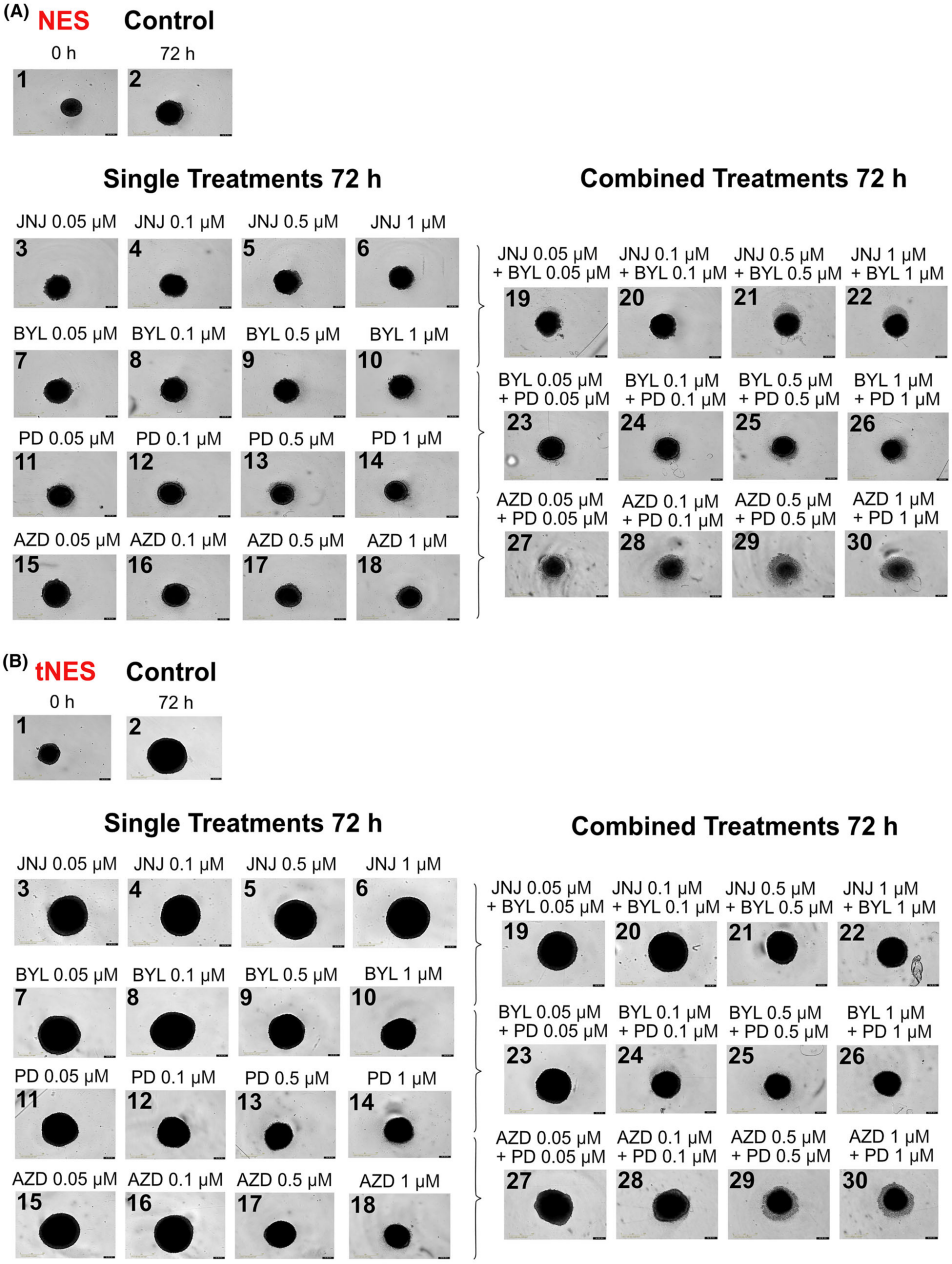
The efficacy of the FGFR, PI3K, CDK4/6, and AKT inhibitors JNJ‐42756493, BYL719, PD‐0332991, and AZD5363, respectively, alone and in combination, on the growth of NES and tNES spheroids. Representative images of 3D NES (A1–30) and 3D tNES (B1–30) are shown 72 h after treatment from three independent biological experiments (*n* = 3). Scalebar = 400 μm. NES, neuroepithelial stem cells; tNES, tumor‐derived neuroepithelial stem cells; FGFR, fibroblast growth factor receptor; PI3K, phosphoinositide 3‐kinase; CDK4/6, cyclin‐dependent kinases 4 and 6; AKT, protein kinase B; JNJ, JNJ‐42756493; BYL, BYL719; PD, PD‐0332991; AZD, AZD5363.

To further explore the effects of JNJ‐42756493, BYL719, AZD5363, and PD‐0332991 administered alone or in combination on 3D NES and 3D tNES in real time, the Incucyte® S3 Live‐Cell Analysis system was used. Graphs presenting spheroid growth up to 72‐h post‐treatment are shown in Fig. 4, and representative images of 72 h after treatments are shown in Fig. 5.

#### 3.4.1 Single treatments

The size of NES spheroids remained mostly unaltered compared to the controls throughout the observation period with all drugs, suggesting little to no visible effect on growth (Figs 4A–D and 5A, 1–18).

In 3D tNES, PD‐0332991 had the strongest effect on growth, leading to the greatest reduction in spheroid size in a dose‐dependent manner (Figs 4J and 5B, 11–14). In addition, the two highest doses (0.5–1 μm) of AZD5363 also decreased spheroid growth, while the same doses of BYL719 had a slight impact compared to the control (Figs 4I,K and 5B, 7–10,15–18).

**Fig. 6:**
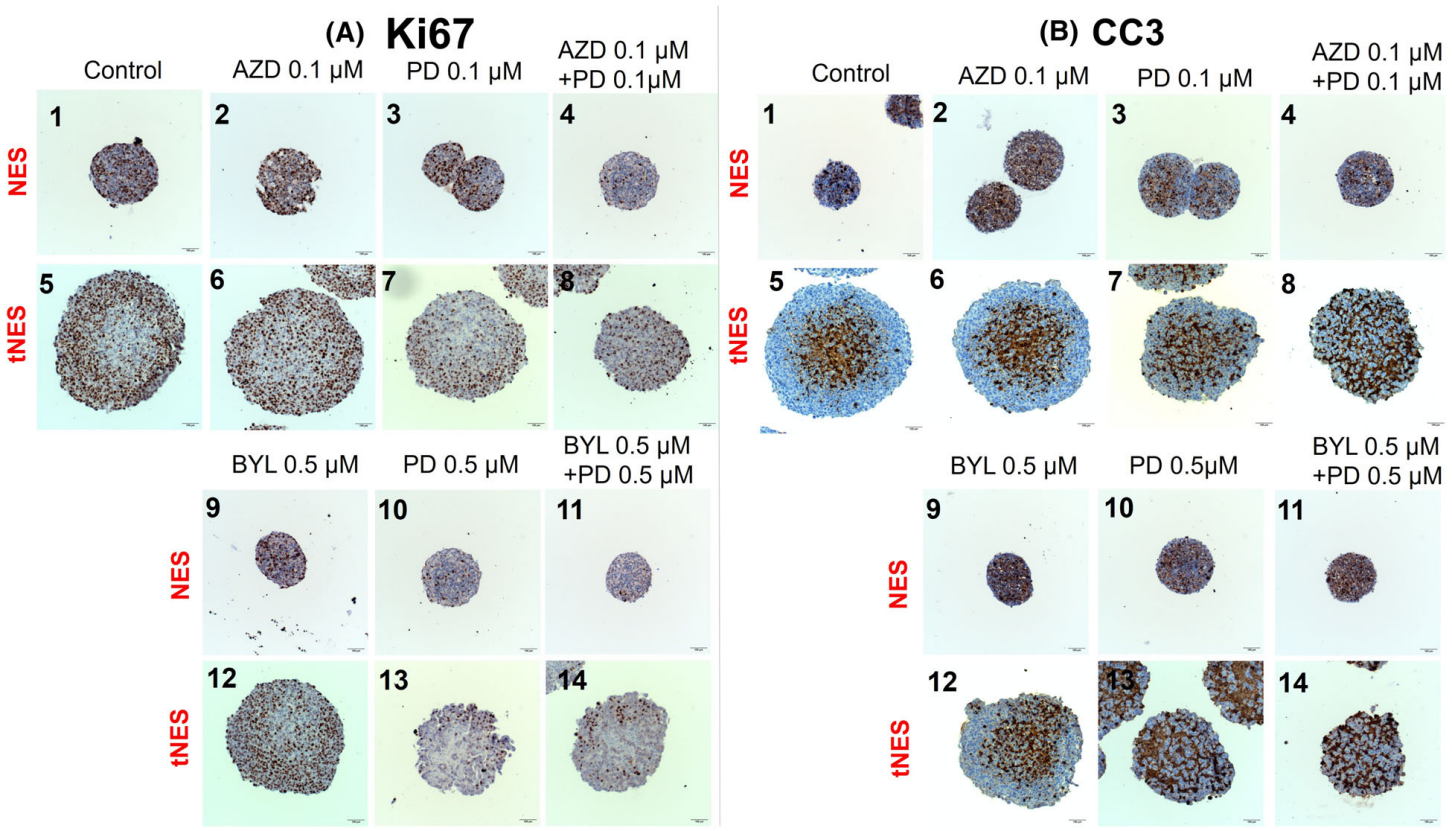
Histological analysis of NES and tNES spheroids 48 h after treatment with single doses of BYL719, PD‐0332991, AZD5363 and their combinations. Representative images of NES and tNES spheroid sections stained for the proliferation marker Ki67 (A) and the apoptosis marker cleaved caspase‐3 (CC‐3) (B) are shown. Ki67 staining is shown for NES (A1–4 and A9–11) and tNES (A5–8 and A12–14), and CC‐3 staining is shown for NES (B1–4 and B9–11) and tNES (B5–8 and B12–14). Images were captured at 20x magnification. Experiments were repeated independently three times (*n* = 3), with similar results. Scalebar = 100 μm. NES, neuroepithelial stem cells; tNES, tumor‐derived neuroepithelial stem cells; BYL, BYL719; PD, PD‐0332991; AZD, AZD5363.

In contrast, JNJ‐42756493 did not affect spheroid size compared to the control (Figs 4H and 5B, 1–6).

#### 3.4.2 Combined treatments

In 3D NES, combinations of BYL719 with PD‐0332991 and AZD5363 with PD‐0332991 had no or minimal impact on growth, with spheroid size remaining largely unchanged (Figs 4E–G and 5A, 19–30). However, the combination of AZD5363 with PD‐0332991 led to the appearance of a halo‐like structure indicative of cellular shedding, suggesting cell dissociation from the main spheroid body (Fig. 5).

In 3D tNES, among combination treatments, BYL719 with PD‐0332991 and AZD5363 with PD‐0332991, profoundly inhibited spheroid growth, with spheroid size decreasing in a concentration‐dependent manner (Figs 4L–N and 5). Similar to 3D NES, the two highest doses of AZD5363 and PD‐0332991 induced spheroid dispersal in tNES spheroids, indicating cellular dissociation (Fig. 5).

### 3.5 Immunohistochemistry (IHC) analysis of PI3K, CDK4/6, and AKT inhibitors on apoptosis and proliferation in 3D NES and 3D tNES

IHC was performed to further investigate the impact of the treatments on proliferation and apoptosis in 3D NES and 3D tNES. Staining was done on 3D NES and 3D tNES 48 h after treatment with efficient single (0.1 μm AZD5363, 0.5 μm BYL719, 0.1 and 0.5 μm PD‐0332991) and combined treatments (0.1 μm AZD5363 with 0.1 μm PD‐0332991 and 0.5 μm BYL719 with 0.5 μm PD‐0332991).

The analysis included markers for proliferation (anti‐Ki67) and apoptosis (anti‐cleaved caspase 3, CC‐3) as shown in Fig. 6A,B respectively.

In the control groups, 3D NES showed moderate Ki67 staining, reflecting normal proliferative activity, while 3D tNES exhibited strong Ki67 staining, indicating high tumor cell proliferation (Fig. 6A, 1,5). However, a notable reduction of Ki67 staining was observed at the center of control 3D tNES, suggesting lower proliferative activity, likely due to nutrient deprivation, hypoxia, or high cell density (Fig. 6A, 5).

3D NES proliferation (Ki67) was affected mainly with single 0.5 μm PD‐0332991 and combined treatments of 0.1 μm AZD5363 and 0.1 μm PD‐0332991 or 0.5 μm BYL719 and 0.5 μm PD‐0332991 as compared to the control, but none of these treatments had a noticeable impact on spheroid size (Fig. 6A, 1–4,9–11).

In 3D tNES, strong inhibitory effects on proliferation (Ki67) were observed with all single treatments (0.1 and 0.5 μm) of PD‐0332991 as well as with combinations of 0.1 μm AZD5363 with 0.1 μm PD‐0332991 or 0.5 μm BYL719 and 0.5 μm PD‐0332991 compared to the control (Fig. 6A, 5–8,12–14). All treatments also led to a noticeable reduction in spheroid size compared to the control, suggesting a considerable impact on both cell division and overall spheroid growth (Fig. 6A, 5–8,12–14).

Control 3D NES and 3D tNES showed minimal CC3 staining, indicating low apoptotic activity (Fig. 6B, 1,5). However, the centers of control tNES spheroids exhibited an increased CC3 staining, suggesting a higher apoptosis due to nutrient deprivation, hypoxia, or cellular stress, consistent with the reduced Ki67 staining (Fig. 6B, 5).

CC3 staining was increased with all treatments in 3D NES (Fig. 6B, 1–4,9–11). Similarly, an enhanced apoptotic response in 3D tNES was observed with all single treatments, though 0.1 μm AZD5363 and combination treatments 0.1 μm AZD5363 with 0.1 μm PD‐0332991 and 0.5 μm BYL719 with 0.5 μm PD‐0332991 all had weaker effects (Fig. 6B, 5–8,12–14). Nevertheless, in general, the effects of the inhibitors were more pronounced in 3D tNES than in 3D NES.

## 4 Discussion

In this study, the effects of single and combination treatments with the FGFR, PI3K, CDK4/6, and AKT inhibitors JNJ‐42756493, BYL719, PD‐0332991, and AZD5363, respectively, on viability and growth were evaluated on 2D and 3D NES and tNES cultures, both derived from a Gorlin syndrome patient SHH‐MB model. Although it is derived from a Gorlin syndrome patient (which is a very limited patient group)— PTCH1 mutations are the most common mutations in SHH‐MB and therefore a valid model system [38, 42, 43].

In general, 2D tNES cells were more sensitive to the inhibitory effects of both single and combined treatments compared to 2D NES cells. Likewise, in the 3D setting, all single‐drug treatments, except for JNJ‐42756493, tended to be more effective in reducing viability in 3D tNES than in 3D NES; this was also the case when combining the drugs, except for combinations including JNJ‐42756493. Notably, JNJ‐42756493 alone or in combination with BYL719 exhibited a very pronounced impact on viability in 3D NES compared to 3D tNES.

The initially observed stronger inhibition of cell viability by most drugs, used alone or in combination, on 2D tNES compared to 2D NES was very promising. It suggested that these drugs could be more effective against the tumorigenic tNES cells than the normal NES cells, which is important for minimizing treatment‐related side effects while effectively targeting tumors. However, upon testing NES and tNES as spheroids revealed a more complex pattern. Although single‐drug treatments again tended to affect 3D tNES more than 3D NES, this was not the case for the FGFR inhibitor JNJ‐42756493. Moreover, the tested inhibitors generally exhibited greater potency in 3D spheroid cultures compared to 2D monolayers. We hypothesize that the differences between 2D and 3D cell viability data could to some extent be due to altered cell–cell interactions, drug penetration, or the presence of a hypoxic core within spheroids.

Notably, combining the drugs, especially BYL719 and PD‐0332991, generally showed synergistic effects on viability for both 3D NES and 3D tNES, while AZD5363 combined with PD‐0332991 showed mainly synergistic effects in 3D NES and predominantly additive effects in 3D tNES. The latter may suggest that AKT signaling might not be as critical in 3D tNES, or the cells can bypass AKT inhibition via other survival routes.

The combination of BYL719 and JNJ‐42756493 was effective in both 3D NES and 3D tNES; however, due to the high sensitivity of NES cells to JNJ‐42756493 alone, this combination was not investigated further. The high sensitivity to JNJ‐42756493 could be a result of the crucial role of FGFR in stem cells, making the signaling pathway essential for survival [44]. This highlights the importance of carefully evaluating FGFR inhibitors in the context of normal brain tissue to avoid potential adverse effects on healthy neural stem cells.

Finally, we also investigated growth, proliferation, and apoptosis in the 3D setting. Overall, the effects were more pronounced on the 3D tNES, which, consistent with their tumorigenic nature, displayed higher proliferative capacity compared to the 3D NES cells. This observation was supported by stronger Ki67 staining in control 3D tNES compared to 3D NES. Further support came from the increased CC3 levels in the core of control 3D tNES after 72 h of growth, suggesting elevated apoptosis likely due to nutrient deprivation, hypoxia, or cellular stress.

Additionally, the tested single and combined inhibitors had minimal effects on 3D NES size, suggesting that 3D NES growth remained mostly unaffected. On the other hand, growth was less prominent in general for 3D NES as compared to 3D tNES during the 72‐h observation period. In contrast, 3D tNES were more responsive, with single treatments of PD‐0332991 and AZD5363, as well as combinations of PD‐0332991 with BYL719 and PD‐0332991 with AZD5363, showing the strongest growth‐inhibitory effects. Notably, single PD‐0332991 and the same combination treatments greatly reduced proliferation and increased apoptosis in 3D tNES, whereas in 3D NES, proliferation was also inhibited by these treatments, but apoptosis induction remained minimal.

The CDK4/6 inhibitor (PD‐0332991) alone or in combination with PI3K (BYL719) or AKT (AZD5363) inhibitors selectively suppressed tumor spheroid growth by both reducing proliferation and increasing apoptosis, whereas in 3D NES they mainly reduce proliferation without triggering cell death or affecting overall growth. This indicates a selective response where tumor cells were more sensitive to both growth arrest and apoptosis, while normal cells mainly show slowed proliferation without much cell death and shrinkage.

This selective response of tNES versus NES cells suggests a potential therapeutic window, in which tumor cells are effectively targeted while sparing normal neural stem cells, potentially reducing treatment‐related side effects in patients. Furthermore, the observed synergistic effects of some combinations indicate that targeting multiple signaling pathways simultaneously may overcome intrinsic or acquired resistance mechanisms in SHH‐driven MB. Clinically, these findings highlight the potential for rational combination therapies that maximize tumor suppression while minimizing harm to healthy brain tissue.

Clearly, the NES/tNES model offers notable advantages, as it allows for the direct comparison of drug responses between a normalized neuroepithelial stem cell line and its tumorigenic counterpart. However, the data should be interpreted with caution, as tNES cells inherently possess a higher growth capacity, which may make them more susceptible to drug‐induced effects overall and this must be considered when interpreting the data.

This study has some limitations. First, it was based on a single NES/tNES cell model derived from one Gorlin patient, which may not fully capture the biological variability seen across different patients. Second, the drug screen included a relatively limited number of drugs and concentrations, primarily focusing on drugs and doses previously tested in MB cell lines [36, 37]. Moreover, this study was restricted to the MB SHH subtype, which comprises approximately 30% of all cases. Future studies should include *in vivo* evaluation using established SHH‐MB mouse models to assess both efficacy and systemic tolerability of these inhibitors. Consequently, the drug responses observed in tNES cells with PTCH1 mutations may not fully reflect responses in MB with other distinct genetic backgrounds.

Nevertheless, the study illustrates how a normalized/tumorigenic NES/tNES model can be used to test various drugs, although the specific features and proliferative capacities of the two counterparts in the model need to be taken into account when interpreting the obtained data.

## 5 Conclusions

To sum up, 2D tNES cells were generally more sensitive to the inhibitory effects of both single and combined treatments compared to 2D NES cells. In the 3D setting, all single‐drug treatments, except for the FGFR inhibitor JNJ‐42756493, exerted stronger effects on cell viability in 3D tNES than in 3D NES, while for JNJ‐42756493 the opposite occurred, and for combined treatments, synergistic/additive effects were relatively similar for both NES and tNES. This NES/tNES model therefore holds promise for evaluating drug responses, but further studies on additional NES/tNES models should be pursued, and the specific cell features and proliferation capacity of the cells should be taken into account when evaluating the data.

## Conflict of interest

The authors declare no conflict of interest.

## Author contributions

ML: Writing – review and editing, writing – original draft, visualization, validation, methodology, investigation, funding acquisition, formal analysis, data curation. MB: writing – review and editing, writing – original draft, visualization, validation, investigation, data curation. PC: writing – review and editing, visualization, validation, investigation, formal analysis, data curation. MW: writing – review and editing, writing – original draft, methodology, visualization, validation. ONK: writing – review and editing, writing – original draft, visualization, validation, supervision, resources, project administration, methodology, investigation, funding acquisition, formal analysis, data curation, conceptualization.

## Data Availability

The data that support the findings of this study are available from the corresponding author [ourania.kostopoulou@ki.se] upon reasonable request.
